# Decentralized Patient-Centric Report and Medical Image Management System Based on Blockchain Technology and the Inter-Planetary File System

**DOI:** 10.3390/ijerph192214641

**Published:** 2022-11-08

**Authors:** Syed Agha Hassnain Mohsan, Abdul Razzaq, Shahbaz Ahmed Khan Ghayyur, Hend Khalid Alkahtani, Nouf Al-Kahtani, Samih M. Mostafa

**Affiliations:** 1 Ocean College, Zhejiang University, Zheda Road 1, Zhoushan 316021, China; 2 Department of Computer Science and Software Engineering, International Islamic University, Islamabad 44000, Pakistan; 3 Department of Information Systems, College of Computer and Information Sciences, Princess Nourah bint Abdulrahman University, Riyadh 11671, Saudi Arabia; 4 Department of Health Information Management and Technology, College of Public Health, Imam Abdulrahman Bin Faisal University, Dammam 31441, Saudi Arabia; 5 Department of Computer Science, Faculty of Computers and Information, South Valley University, Qena 83523, Egypt

**Keywords:** digital health, blockchain, smart contract, Ethereum, distributed storage, IPFS, medical images sharing, health system

## Abstract

Several academicians have been actively contributing to establishing a practical solution to storing and distributing medical images and test reports in the research domain of health care in recent years. Current procedures mainly rely on cloud-assisted centralized data centers, which raise maintenance expenditure, necessitate a large amount of storage space, and raise privacy concerns when exchanging data across a network. As a result, it is critically essential to provide a framework that allows for the efficient exchange and storage of large amounts of medical data in a secure setting. In this research, we describe a unique proof-of-concept architecture for a distributed patient-centric test report and image management (PCRIM) system that aims to facilitate patient privacy and control without the need for a centralized infrastructure. We used an Ethereum blockchain and a distributed file system technology called the Inter-Planetary File System in this system (IPFS). Then, to secure a distributed and trustworthy access control policy, we designed an Ethereum smart contract termed the patient-centric access control protocol. The IPFS allows for the decentralized storage of medical metadata, such as images, with worldwide accessibility. We demonstrate how the PCRIM system design enables hospitals, patients, and image requestors to obtain patient-centric data in a distributed and secure manner. Finally, we tested the proposed framework in the Windows environment by deploying a smart contract prototype on an Ethereum TESTNET blockchain. The findings of the study indicate that the proposed strategy is both efficient and practicable.

## 1. Introduction

The introduction of information technology (IT) has made an immense contribution towards improving the overall health of patients around the world as it allows for efficient service delivery. Patient information systems enable the easy tracking of individual health challenges and treatment over time, giving insight into the diagnosis and treatment of patients. There is notable evidence associated with the use of advanced clinical information systems to significantly enhance the safety, quality, and patient-centeredness of care [1]. Due to fragmented information production and storage, personal health information is not fully exploited to promote effective and efficient treatment, making data management cumbersome and overall safety open to compromise. Within hospitals, medical clinics, laboratories, and pharmacies, health information systems are often separated. Although information collection, processing, delivery, and management are significant to healthcare delivery, the healthcare industry has historically trailed far behind, compared to other industries, in terms of investments in both data delivery and communication technologies [2]. The role of information technology (IT) permeates the medical industry of the modern era. Beginning in the 1970s with the integration of hospital information systems (HIS), digital imaging modalities, including computed tomography (CT) in the 1970s and magnetic resonance imaging (MRI) in the 1980s, picture archiving and communication systems (PACSs) in the 1980s, and softcopy reading in the 1990s, have evolved into today’s computerized exchange of healthcare data across regions, countries, and even globally. The internet has evolved into a valuable source of knowledge and a cost-effective way of communication. However, the ongoing expanding role of the internet and IT has raised new technical challenges, and one topic that has become of substantial interest for hospitals is cybersecurity, which is “the state of being protected against the criminal or unauthorized use of electronic data”.

The move to electronic health record management has prompted the usage of various new acronyms by practitioners and their patients, including electronic medical records (EMRs), personal health records (PHRs), and electronic health records (EHRs) [3]. Medical images and patient information, including physician names, personal statistics (e.g., weight and age), data from home monitoring devices, and other text-based data processed by practitioners, are typically included in these health records. Even though they are connected to the same patient, medical photographs and patient information are saved and kept by separate hospitals.

Large amounts of data are processed and preserved by cloud servers in their capacity as distributed nodes for data management and processing. One of the potential concerns associated with a centralized authority to process and manage data, such as cloud servers, is single-point failure [4]. To prevent such a catastrophe, all third parties contribute to cloud storage [4]. A blockchain is an impenetrable, decentralized digital transaction ledger. By doing away with the requirement for a third-party middleman, blockchain technology offers confidence and transparency in the development of a trust-enabled paradigm [5]. Decentralized storage, commonly referred to as distributed ledger storage, is a feature of blockchain-based solutions that enables users to store their data on other network nodes autonomously. The issue is that network nodes have a finite amount of processing and storage capacity [6,7].

Hospitals will need more storage capacity in the future to analyze such a massive amount of image data [8,9], and the demand for sensitive images will increase rapidly. As a result, it is critical to securely store and communicate medical images without interruption. Due to privacy, accessibility, storage, and security problems, the current technologies for transporting medical images and patient information that are implemented in centralized data centers have been determined to be unsuitable. The potential breaches of medical record data within major medical data centers have posed significant challenges for any organization developing medical image processing systems in recent decades [10].

As a result, a patient-centric system is required, in which a patient has complete control over their medical images and can trace them online without any dependency on a centralized framework. The move toward decentralized technologies such as blockchain [11] has become an important trend that has the ability to create a novel groundwork for reducing the above-discussed challenging issues and encouraging the more general adoption of a patient-centric system.

Medical images are several orders of magnitude greater than those available on a public blockchain [12]. The Protocol Labs devised the Inter-Planetary File System as a distributed web to answer the challenge of decentralized storage (IPFS) [13]. The IPFS was created to allow for the sharing and storage of hypermedia in a distributed file system using a content-addressable, peer-to-peer (P2P) technology. Through the support of off-chain storage capability, the IPFS offers the unique benefit of interoperability with several blockchain networks. The IPFS delivers distributed data access systems with persistent, smarter, and quicker online services.

However, there are significant barriers to storing sensitive medical images via these distributed storage options, including unwanted access and patient image privacy issues. It is critical, for example, to be able to handle large amounts of data between general practitioners, hospitals, patients, and medical institutions while minimizing the risk of privacy breaches. Another key feature of a secure and private storage system is its capacity to lower the expense and limitations of medical image gathering by removing the requirement for centralized parties [14].

**Figure 1:**
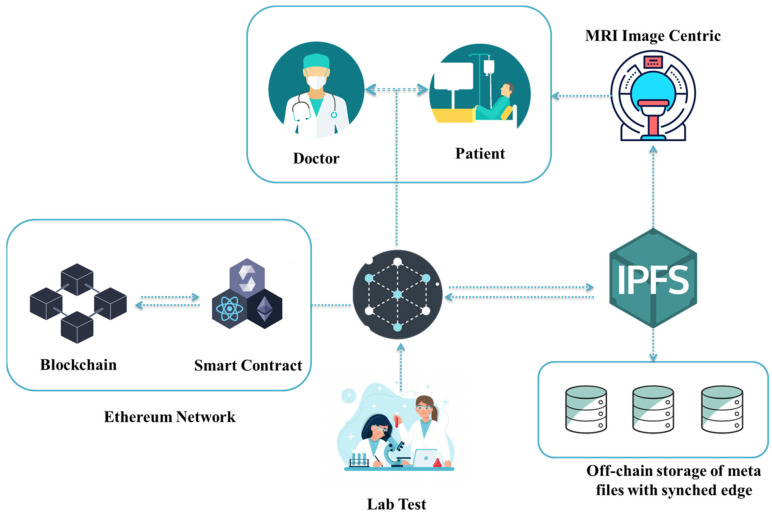
Overview of the proposed solution.

An illustration of the entire system is shown in Figure 1. This system displays every single process. The user must be authorized before utilizing this system; users include doctors, patients, and anyone taking lab tests. In the registration procedure, users request permission to use this system. The user, who has taken a lab test, can upload the test results and MRI radiologist image data through the site after receiving authorization. Only patients or doctors who have been given permission may access the report’s data.

To respond to this question, a proof-of-concept (POC) design is proposed for a distributed architecture named the patient-centric image management (PCRIM) system, which is a blockchain-assisted framework developed to enable the storage of encrypted medical images and secure patient-centric access within a public distributed network. The contributions of this research can be:

- To give a high-level overview of the proposed PCRIM system’s structure and show how the system’s various components interact.
- To give access to doctors and patients to the medical test reports, we offer a patient-centric access control system based on a smart contract. Specific functions are used to send data into and out of the Ethereum blockchain as well as to provide access privileges between parties.
- We use architecture to see if the notion is feasible. In order to do this, we built a patient-centric control system prototype on the Ethereum test network. The associated source codes have been made available on the internet.
- We used test cases to validate functionality and evaluated the proposed framework’s capabilities by considering the following performance metrics: image and test report access time, time needed to record system events in the blockchain, cost of running functions, average gas consumption, and average block size.

The remainder of the article is arranged as follows: Section 2 discusses the background and current state of the art. Section 3 describes the research methodology and motivation consequence. Section 4 discusses the implementation of technologies and methods as well as the verification and analysis of the proposed system. The evaluation and validity threats are presented in Section 5. Finally, Section 6 brings the process to a close with a conclusion.

## 2. Legacy Evolutions: Existing View and Emerging Technical Challenges

### 2.1. Health Care Medical Image System

The current reality of sharing health information is slightly uncomfortable for those of us who do not have a clear idea of the future use of that information. Imaging departments present a difficulty that goes beyond what transferring patient documents implies.

The information age has arrived. As digitalization and cloud storage become increasingly common, an extensive amount of data is being moved from paper to electronic devices [15]. In medical institutes [16], patient information is frequently digitized and stored. A private database is often used to record electronic medical reports, which creates a challenging issue when patient data are dispersed among several hospitals as a result of life events that lead these patients to move from one hospital to another. These records are created at hospitals after patients’ visits and are recorded in electronic medical records. As a result, even though the data belong to them, the patients lose simple access to their records [17]. They are unable to present their detailed prior medical records to doctors when they visit other hospitals since their data are stored elsewhere. Data exchange is hampered by interoperability issues across different healthcare systems. Due to a lack of consistent data management and sharing, it is hard for people to acquire the data they require.

Blockchain, a distributed digital ledger, is both tamper-evident and tamper-resistant, typically without the use of a central repository or authority (e.g., a government, company, or bank). At the basic level, it permits a user community to store transactions in a shared ledger inside that user community, ensuring that under typical blockchain network operation, no transaction can be modified once published. Modern cryptocurrencies, which are electronic forms of payment secured by cryptographic processes rather than an authority or central repository, were first developed in 2008 when the blockchain concept was initially incorporated with several innovative technologies and computer ideas [18].

Despite the wide range of blockchain network variations and the quick advancement of new blockchain-related technology, the majority of blockchain networks adhere to similar fundamental principles. Blocks make up a distributed ledger known as a blockchain. Each block is comprised of a block header that holds information about the block and a collection of transactions as well as block data with additional information. Every block header on the blockchain (except the very first block) has a cryptographic link to the header of the block before it. Each transaction is digitally signed by the entity or user who sends it and involves one or more users of the blockchain network as well as a record of what has happened [18].

Blockchain’s special qualities are motivating researchers to seek its larger-scale implementation. Transmitting data is made possible by a blockchain, which offers a secure, reliable, and trustworthy platform [19]. As a result, it provides traceability by recording any unauthorized data access. The ability of networks to control themselves is compromised by their dispersed structure. Additionally, a significant network attack is a possibility due to the immutability of blockchain [20].

### 2.2. Challenges to Health Care Systems

In Health Level 7 (HL7), transmitting test results, operation notes, and other items involves mostly text and information presented in the form of tables [21]. These elements, as well as images, are generally sent in the Digital Imaging and Communications in Medicine (DICOM) format in radiology records. The following interaction situations, in order of increasing complexity, are typical for both radiologists and clinicians.

On one hand, data requestors look for the historical medical records of patients in order to develop their treatment plans [22,23]. Medical records held in private databases, on the other hand, include a great deal of information about the institution and the patient. As a result, requesting and sharing data may put data providers’ confidentiality at risk. EMRs are not accessible to everyone. Several academics have offered several relevant schemes concerning cloud storage and computational technologies to give adequate solutions to compress storage and process requirements in order to fulfill the increased demands on data sharing [24]. However, cloud service providers (CSPs) encounter major challenges concerning hospitals when employing centralized cloud services because of the risks associated with the exposure of data. To address the issues with medical data exchange, certain cryptographic approaches have been developed. However, they are inadequate; disadvantages continue to exist [25]. The huge amount of data stored by other parties is an uncomfortable security risk for the hospital [26].

Personal medical data is protected under privacy rules, and several legal measures make it illegal to keep personal data indefinitely. Hospitals have no motivation to offer the data to third parties as they wish to avoid potential legal issues due to data leaks.

Medical records [27] must be monitored by the government in order to uncover illegal medical operations. Meanwhile, experts expect to achieve a breakthrough in the identification of new approaches and cures for healing illnesses by analyzing historical medical data [28]. “Healthcare rallies for blockchains: Keeping patients in the center” is a whitepaper published by IBM’s Institute for Business Value [29]. According to the report, blockchain technology will be utilized for managing clinical trial records and monitoring compliance, and electronic health records (EHRs). At present, several government bodies have devoted their efforts to this technology. For instance, a blockchain industrial park has been established by the Chinese government in Hangzhou city, with the goal of allowing more organizations and businesses to benefit from blockchain technology in a variety of fields.

### 2.3. Related Work

The approach of registering and exchanging medical health records has evolved dramatically over the last 20 years due to tight practical standardizations, the utility of complicated technology, and precise diagnosis and treatment. Medical images are routinely exchanged on DVDs or CDs sent between patients, physicians, and hospitals in order to get a diagnosis. However, using this technology may result in medical images being damaged or intercepted because of patient or physician mistakes [30].

Web server vulnerabilities, cross-site scripting, and default accounts, according to the researchers [31], might lead to PACS access breaches and irreversible medical image alterations. Additionally, current storage and access-sharing models have revealed a number of issues, including privacy concerns arising from the central repository’s storage of patient identifiers, image ownership managed by the officials, and the mismatch of patients in the registered healthcare database [32]. As a result, it is essential to develop a decentralized interoperability system that takes into account a decentralized framework while also enabling privacy options, electronic consent, authentication, security protocols, and data provenance.

Several researchers have recently worked on establishing a framework for medical health record access sharing that combines a cloud service and a blockchain [33,34]. Patients can use a private digital key to access encrypted photographs and selectively share medical records. The authors explored the idea of permitting the machine learning (ML) approach to access several images stored on the blockchain network in order to drive the optimization of computer-aided statistical analysis, but cost-effectiveness and scalability must be addressed before this technology is standardized. A study created a breadcrumb system for a MedBlock medical record search [35]. Breadcrumbs were used to keep track of the addresses of blocks holding patient data. However, because of the expanding increase in fragmented data, these solutions are not suitable for the process of finding data through a blockchain.

MeDShare, a hybrid cloud-based sharing mechanism for EHRs empowered by a centralized cloud server provider, was presented by the authors of [33]. A later development used two decentralized networks, known as MedChain [34], as the external server. The authors developed a session-enabled data-sharing solution and a digest chain framework utilizing an immutable blockchain and a modifiable P2P storage structure in the MedChain idea. The modifiable P2P storage architecture, on the other hand, puts the possibility of altering and modifying stored patient health information at risk.

For secure data storage and access control management, Zyskind et al. advocated for blockchain technology [36]. According to this study, encrypted data are stored with trusted third-party hosting providers, and events can be logged on the blockchain. In the real world, there are no reliable third parties, as blockchain technology poses critical concerns regarding data leakage.

Xia et al. [33] presented a method for properly managing and protecting medical records. The solution is blockchain-based, and it protects and manages shared medical data in cloud repositories across big data companies. It protects data by confirming the cryptographic keys and authenticity of identities. However, the approach does not address issues regarding data leakage. That is the major reason that hospitals are hesitant to share data with a third party, making the system unworkable.

Through the integration of clinical data, certain prognostic models have recently been suggested, the majority of which are based on ML techniques [33]. Instead of exploring clinical pathways, they concentrate on the incorporation and prediction of the medical state. Additionally, privacy protection is not taken into account. However, the integration merits attention. The distributed fractional knapsack problem was proposed with privacy-preserving optimization [37,38].

Before uploading medical images to a vast IPFS network, Jabarulla et al. [39] secured them using an encryption technique to guard against illegal access. Swapping encryption keys allows the users to securely access private medical images. However, it does not provide the lab report features for different types of blood tests.

Both traditional and blockchain techniques mostly rely on centralized infrastructure and need the storage and access of medical images by a trusted third-party organization. In this paper, we present a solution for achieving secure patient-centric access in a decentralized architecture system, empowered by the notion that patients should own their medical images. We demonstrate the viability of combining the IPFS and blockchain for storing and accessing patient medical images. We carried out a small-scale experiment by designing an architecture system to evaluate system operation, considering test cases, and analyzing the suggested framework’s potential, enabled by transaction efficiency, cost, and image access time.

Table 1 compares the proposed framework to competing blockchain-based medical health record management systems in a succinct manner. It is clear from this table that the suggested PCRIM system has more benefits than the alternatives that are currently available. Several studies [28,29,30] also considered centralized architectures in which the failure of a single central node results in the failure of the entire system. In contrast, the structure suggested in this research has separate nodes, ensuring reliable and effective data access.

## 3. Research Methodology and Motivation Consequence

**Figure 2:**
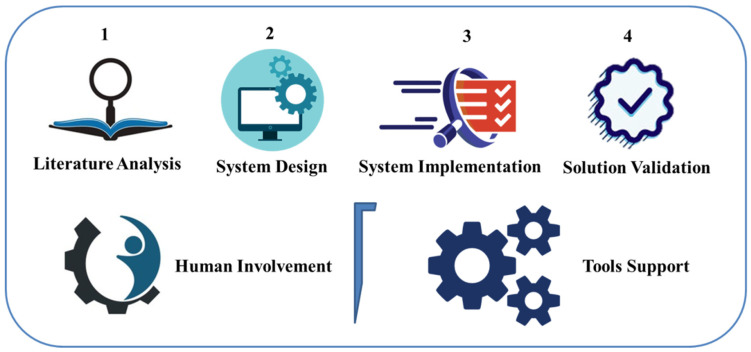
Overview of the research methodology.

We now describe the study approach, which contains the design specifications for the suggested solution. A summary of the proposed research technique is depicted in Figure 2, which includes four processes that follow an incremental mechanism to assess, develop, execute, and validate the solution, as presented below.

Step 1—The first stage is to undertake a critical study of an extensive variety of current literature (for example, peer-reviewed published articles, technical reports, technological road maps, and so on) [40,41] with the aim of identifying current solutions and their limitations. We reviewed the most relevant and current papers, as per the suggestions, to perform a systematic literature review [42]. By examining existing research and development solutions, we streamlined the needed approach and defined the scope of this study.

Step 2—The design of software systems is all about modeling the solution before it is implemented during the development phase of a methodology. We followed certain standards and suggestions to simulate systems in order to design the proposed solution; we used the ISO/IEC/IEEE 42010:2011 standard to design our solution [43].

Step 3—The execution of an approach in the shape of computational and storage-intensive phases is what algorithm implementation includes. The algorithmic solution is a modular deconstruction of an approach that users may adjust with specified inputs. Design executable standards are the products of algorithmic complexity and underlying source codes (see Section 4 of the algorithms for more information).

Step 4—The validation of the solution, the final phase, assesses the functionality and quality of the offered solution. We utilized the ISO/IEC-9126 [44] model in methodology to measure system quality. Using a set of well-established assessment metrics, we focus on evaluating a range of elements of system efficiency and usability (see Section 5 of the evaluation).

The first two processes, as shown in Figure 2, are completely manual jobs that require human intellect and decision assistance to execute. To (semi-)automate solution development, the remaining two phases, on the other hand, require human interaction and tool support. For example, solution validation may require algorithm improvement to increase efficiency or modify functionality.

**Figure 3:**
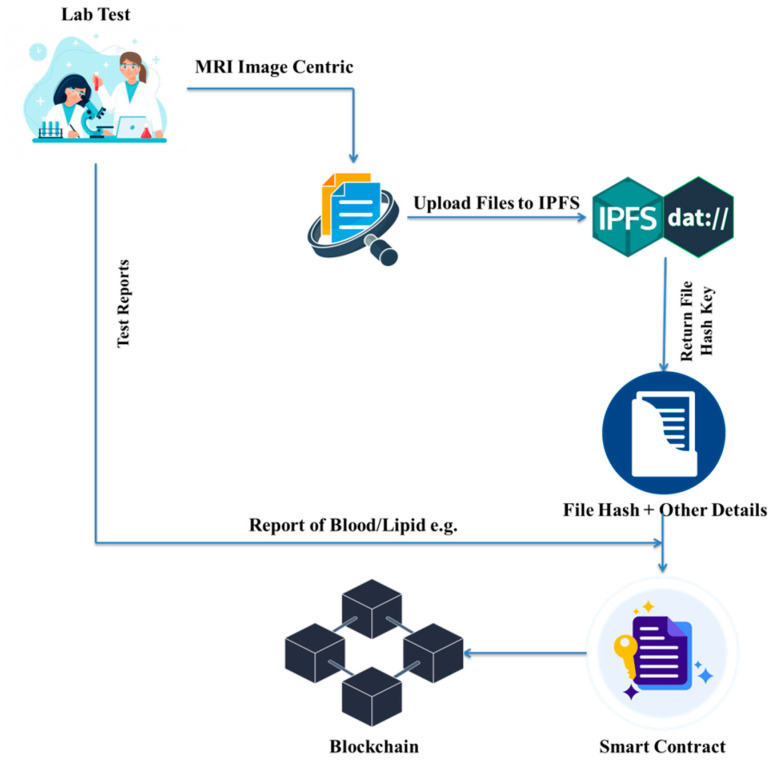
Data storing process.

Figure 3 presents the process of storing medical images and test reports. A test report is a piece of textual information that is inserted by a lab assistant into the blockchain directly using a smart contract. MRI medical images are stored in the IPFS by the radiology part of a lab. In medical image uploading, the radiologist uploads the available medical image data to the IPFS and receives the hash key that is stored in the blockchain with the other required information. Both execution processes are different; test reports could be blood test reports or lipid test reports, for example, but the medical image is a separate part that is stored in the IPFS; then, its file hash is mapped with other required details to be stored in the blockchain.

The digital data-sharing process begins with the creation of metadata for the original file. The medical image metadata comprise information such as the file’s name, size, description, and type. When the metadata are completed, the data are uploaded to the IPFS with the data file. An example of a file upload to the IPFS is as follows:

As per the above function of “AddLipidTest”, we send the required parameters to store the data in the blockchain through a smart contract. This function is written in the smart contract using the Solidity language. We mapped three different mapping sets for searching the data on the portal. The first category is used to get a list of all lipid tests, the second mapping is used to get data by appointment ID, and the final third mapping is set with the patient’s ID and the patient’s appointment ID to access the data for a patient.

We submit the appropriate parameters to save the data in the blockchain through a smart contract, utilizing the “AddBloodGroupingRh” function. To find data on the site, we created three categories. The first category is used to acquire a list of all blood tests, the second mapping is used to get data by appointment ID, and the third mapping is used to access data for patients by patient ID and patients’ appointment ID. Then, we launch the “BloodGroupingRhCreated” event.

We use a smart contract to save the data into the blockchain and the “AddImageCentric” function to provide the relevant parameters. We divided the mapping into two groups. The first mapping is used to get a list of all blood tests based on the patient’s appointment ID, while the second mapping is used to get data for patients based on their patient ID and appointment ID. Then, we launch the “ImageCentricCreated” event.

**Figure 4:**
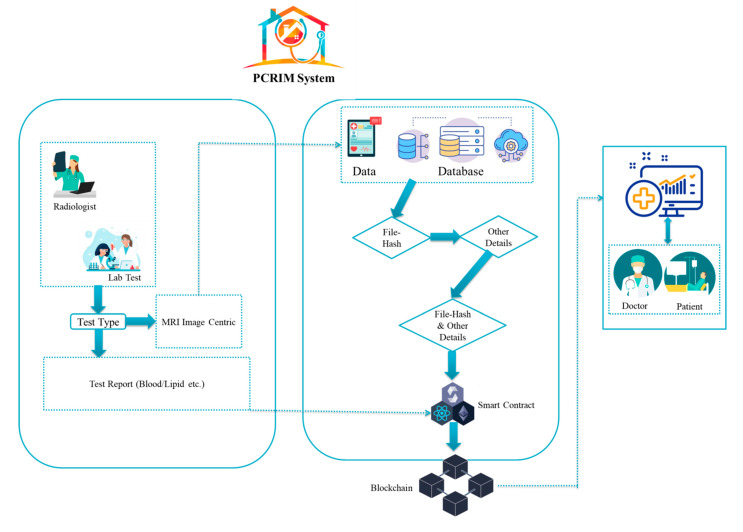
An illustration of the detailed solution.

Figure 4 shows two sorts of medical data uploading categories in DApp. The first are the test reports of patients that are handled by a lab. The lab assistant takes the sample of the patient’s blood and runs the test for the required report. The lab assistant stores the record in the blockchain against the patient ID and appointment id. A radiologist connects the medical image to other examinations and tests and then uploads the medical image to the IPFS using the DApp system and provides the rest of the required details against the patient’s ID and appointment ID, which are stored in the blockchain. This service cycle of events continually repeats itself after a fixed time or is triggered by receiving medical image data from the server.

The dashboard, which is for accessing the available data publicly, provides the data to both the user-doctor and the patient. It is an open-access platform that allows users to view and access medical reports and medical images, which can be downloaded. The medical images and test reports may be accessed via a web portal. The user is allowed to access the data against the patient ID and with the mapping of the appointment ID.

## 4. Implementation of Algorithms and Technology for a Solution

In this section, details on how to implement the solution are supplied. The suggested system is an Ethereum-based blockchain-enabled private network. We considered Ethereum as it is a distributed open-source platform that makes good use of Solidity.

### 4.1. Overview of System

Visual Studio Code: Visual Studio Code (VSC) is a cross-platform code editor by Microsoft that works on a range of operating systems. VSC is a dual-licensed source-code editor for Windows, Linux, and macOS from Microsoft. Debugging tools, highlighted syntax, intelligent code completion, integrated Git control, and code rewriting are all available [45].

Ganache: Ganache is a blockchain-based emulator that can conduct a variety of tests and commands. Ganache is a personal Ethereum blockchain that can be used to run tests, deploy contracts, and construct apps. It inspects the system’s statuses and, thereby, controls the blockchain’s functioning. It was once called Test RPC, but it was later renamed Ganache [46].

Metamask: Metamask refers to a browser extension that connects to a distributed web. Rather than operating the entire Ethereum node, it runs Ethereum decentralized apps in the browser. To access their Ethereum wallet, users can utilize a browser [47,48].

IPFS: IPFS is a decentralized open storage system that uses a hash string route to move data. It is used to hold data that has been encrypted and contains additional data. The routes operate in a likewise fashion to the traditional web’s universal resource locator. As a result, all medical data may be retrieved using their hash at any moment.

Contract Creating: This function is created and executed only by a lab assistant, radiologist, doctor, or patient to upload and store medical data and for accessing the data.

In blockchain systems, the general algorithm known as Algorithm 1 is always used by the blockchain address. However, we also entice the user by making the algorithm’s conditions easy to grasp. We execute the main function with the list of parameters after verifying the address.
**Algorithm 1** Contract Creating1: Input: L (parameters)List of Parameters 2: Output: bool
3: procedure SmartContract
4:    if msg.sender is not Φp then Get address to execute the smart contract 5:       throw; 
6:    end if 
7:    Function Execution (L parameters)Execute the required Function 8: end procedure


In Algorithm 2, the uploading feature for medical data is demonstrated and described. The technique is utilized for uploading the data to the IPFS and saving the file hash in a smart contract with the mapping of certain additional properties. There are many parameters that are linked to the submitted data’s file hash (User ID, Appointment ID, Description, Date).

- Input(s): The parameters are mapped with a file hash key using the algorithm’s input.
- Processing: The medical data image file is read and transformed into a buffer package, which is then posted to the IPFS as a medical data file, and the hash key is returned. Additional parameters are linked to the hash key of the submitted data. User ID, Appointment ID, Description, and Date are entered into a smart contract and kept on the blockchain.
- Output: The mapped data are stored in the blockchain as the output.

**Algorithm 2** Uploading Image-Centric Data1: Input: ∪(ιd),∂(ιd), ∆p, γ℘User ID, Appointment ID, Description, File2: Output: RReturning Result3: procedure DataCentricModuleEvent based function 4:     if User = Lσβ thenUploading by User OR System5:       FS ←  File(γ℘) Get File stream FS 6:       FB ← Buffer.form (FS)Convert FS to Buffer FB7:       FH ← IPFS.Add (FB)Get Hash of Uploaded Data FH 8:       R ← SBC(∪(ιd),∂(ιd), ∆p, FH) Store Data to Blockchain with file hash 9:    end if 
10: end procedure


In Algorithm 3, the medical reports stored in the blockchain ledger feature are demonstrated and described. The technique is used to store medical reports such as blood tests or lipid test reports, for example, to a blockchain ledger using a smart contract with a mapping of certain additional properties. There are many parameters that are stored in the blockchain, e.g., the parameters for lipid tests (Patient User ID, Prescription ID, Cholesterol HDL, Cholesterol LDL, Triglycerides, Total Cholesterol LDL HDL ratio, Appointment ID) and the parameters for a blood test (Patient User ID, Prescription ID, Blood Group, Appointment ID).

- Input(s): Using the algorithm’s input, the parameters are translated to user ID and user appointment ID.
- Processing: The medical data is subsequently submitted as a medical data report to the blockchain ledger. For preserving data in the blockchain, additional factors such as user ID and user appointment ID are connected. User IDs and appointment IDs are translated to the test report parameters and saved in a smart contract on the blockchain.
- Output: The mapped data are stored in the blockchain ledger as the output.

**Algorithm 3** Blood & Lipid Test1: Input: B (parameters), L (parameters), τList of Parameters (Blood/Lipid), Test Type2: procedure Blood-LipidTest Event based function3:    if τ == B || τ == L thenTest Type4:       if τ == B then
5:         µ ← Blood(B (parameters))Get Data of Blood Test6:       end if
7:       if τ == L then
8:         µ ← Lipid(L (parameters))Get Data of Lipid Test9:       end if
10:    end if
11:    R ← Save(µ)Execute Smart Contract To Add Test Record in Blockchain12: end procedure


Algorithm 4 validates the data accessing capabilities, which are then given in this section. The algorithm is used to obtain data from the blockchain and make it publicly visible. The data from the blockchain may be accessed by the user based on the parameters set. There are several sorts of data access; for example, a user can access data based on their user ID and appointment ID mapping. A doctor can access the medical report directly by user appointment ID.

- Input(s): The settings for accessing the data are mapped using the algorithm’s input.
- Processing: The data from the blockchain might be accessible in a variety of ways, such as by a user ID mapped to an appointment ID or by a doctor accessing medical reports using a user appointment ID.
- Output: The result is publicly accessible data that has been mapped.

**Algorithm 4** Interface Layer1: Input: U(ιd), a(ιd), U(τ), τUser ID, Appointment ID, User Type, Test Type2: Output: RDisplay analytics3: procedure InterfaceModuleEvent based function4:    if U(τ) == D thenDoctor To Check Report5:       if τ == B || τ == L  thenTest Type Blood OR Lipid6:         µ ← GetReport(a(ιd)) Return Test Report7:       end if
8:    else
9:       if τ == B || τ == L thenTest Type Blood OR Lipid10:         µ ← GetReport(U(ιd), a(ιd))Return Test Report Map with User ID11:        end if 
12:     end if
13:    R  ← UpdateDashboard(µ)Show Data on User Screen14: end procedure


### 4.2. Tools and Technologies for Algorithmic Implementation

**Figure 5:**
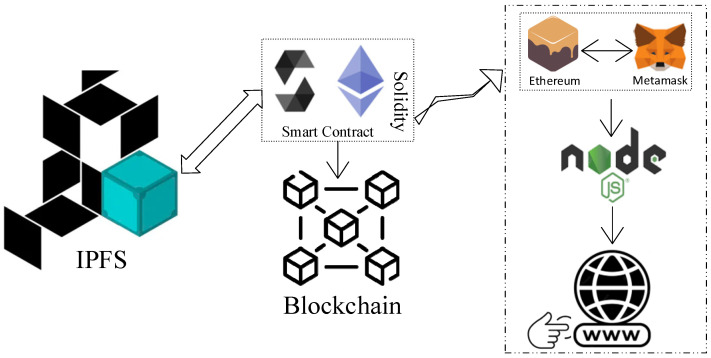
An illustration of technologies and tools for system implementation.

The complementary function of relevant tools and technology for the suggested solution is summarized in this section. The purpose of this discussion is to empower the reader with better knowledge of technology. As seen in Figure 5, the tools and technologies are stacked. If the portal user is a radiologist, the data may be a medical image file that is submitted in an encrypted form to the IPFS platform and returned as a hash key. A server-side application is built using the NodeJS platform, which contains a number of tools. To launch the NodeJS application, we used Visual Studio Code (VSC). To construct a local blockchain environment, we used the Ganache Truffle Suite package to quickly establish a personal Ethereum blockchain that can be used to run tests, issue commands, and observe patient states while managing the operations of the chain.

## 5. Evaluation and Validity Threats

In this part, the results of the recommended solution are reported. We first present the assessment environment, followed by a fuel-usage-based evaluation of smart contract functionality. Following that, we use criteria to assess and quantify data uploading and storage to the blockchain, to query responses such as performance, and to assess the efficiency of algorithmic execution. The assessment criteria are based on the ISO/IEC-9126 model, which is intended to assess software-intensive systems’ quality. Threats to the research’s validity and any limitations that must be addressed are also discussed.

### 5.1. Evaluation Environment

The evaluation environment is a collection of software and hardware and resources for running the solution and tracking different execution phases and outcomes. On the hardware side, evaluation experiments were carried out on the Windows platform by a radiologist uploading lab test findings and medical images to the IPFS (core i7 with 16 GB of runtime memory). In the software industry, execution evaluation, often regarded as evaluation scripts, automates system testing. Such scripts are written in NodeJS and run in Visual Studio Code using the ReactJS language. Several existing libraries, including some of those in development process (react, web3, ipfs.http). For instance, during the upload of medical data images to the IPFS and data storage in the blockchain as well as the retrieval of data from the blockchain, a JavaScript performance library script is used to analyze CPU consumption. The Ganache suit is used to create a local Ethereum blockchain environment, and the Metamask extension is used in the browser to link the distributed web. The Metamask plugin links local Ethereum accounts to the Ganache suit, which then uses the gas transaction cost to perform system functions.

### 5.2. Data Uploading and Fuel Consumption

In order to execute the Ethereum smart contract, the fuel must be paid for. As a consequence, the fuel consumption during the original data transfer was calculated and a comparison was performed for the fuel consumption during the proposed data upload. Gwei is the smallest unit of Ether, the Ethereum cryptocurrency, and it is utilized to track fuel use; 10^9^ wei are known as Gwei.

The cost of contract migration execution was specified in our suggested solution (shown in Table 2). The price is given in Ether, and the gas used is noted. Ether is equal to the amount of gas utilized multiplied by the price of gas. The gas reflects the continual computing cost in this system. The gas price is changed by the network [34] to account for alterations in the value of Ether.

We established a gas restriction by default in the implemented prototype of our system. A contract is created once at the cost of 0.05738454 Ether, with a total gas use of 2,869,227. The migration necessitates contract formation at a low cost of 0.0054726 (Ether) and gas consumption of just 27,363. If the amount of the input data is kept to a minimum, the overall expenses can be further reduced. These expenses, however, are less than those involved in renting storage space from a third-party provider or managing a database utilizing a centralized system such as MedChain [34].

**Figure 6:**
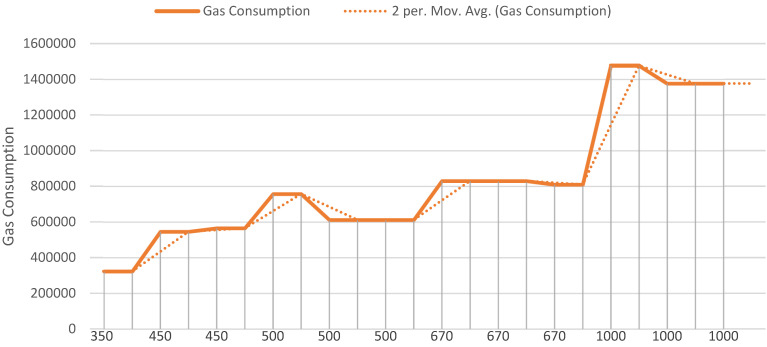
Gas utility based on transaction count and block size.

The time it took for users to upload and store data to the IPFS and blockchain ledger was the final test item. The overall time spent uploading medical data, recalling available data, and reviewing data is referred to as the data uploading and accessing time. Figure 6 shows the outcomes of a series of experiments with average data sizes. While uploading data of 450 bytes, the average fuel consumption was around 555,062 Gas, and when storing data of 1000 bytes, the average fuel consumption was around 1,409,568 Gas. This demonstrates that as the size of the data grows, so does the amount of gasoline consumed. However, even though the data amount increased, there was no significant difference in fuel use when the medical data were uploaded to the IPFS using the suggested methodology.

**Figure 7:**
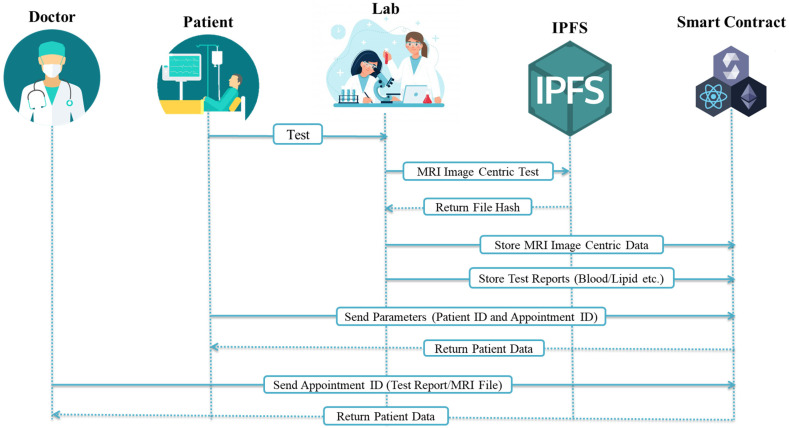
Data-sharing sequence process.

The sequence diagram for all of the network’s entities and their interactions is shown in Figure 7. The system’s execution flow is visualized in Figure 7 to show how it works. There are five entities (Doctor, Patient, Lab, IPFS, and Smart Contract). Doctor and patient entities are used to access the data from the blockchain ledger using the web portal. The lab entity is considered for uploading medical images to the IPFS and getting back the file hash, which is mapped with other required info parameters and stored in the blockchain.

### 5.3. Evaluations of Query Response Time

**Figure 8:**
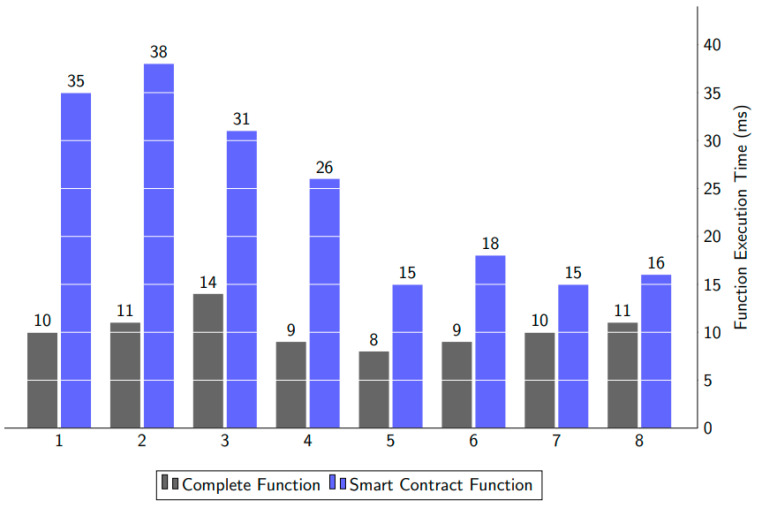
Computational time for function execution to store the data in the IPFS and blockchain.

For saving a medical image to the IPFS and keeping the records’ information in the blockchain, data querying is needed. The query response time may be considered to evaluate a solution’s ability to store and retrieve data from a blockchain. We ran two distinct tests: one for the query response time to store medical image data to the IPFS and another for the query response time for saving records with file hashes to the blockchain. Figure 8 represents the result of the query response time in milliseconds, with the horizontal axis showing the two execution functions and the vertical axis representing the response time in milliseconds. The “Complete” function shows the execution of the entire process, from storing the medical image to the IPFS to saving the record information to the blockchain with the medical data file hash. The “Smart Contract” function displays the delay caused by Metamask’s Smart Contract execution call.

**Figure 9:**
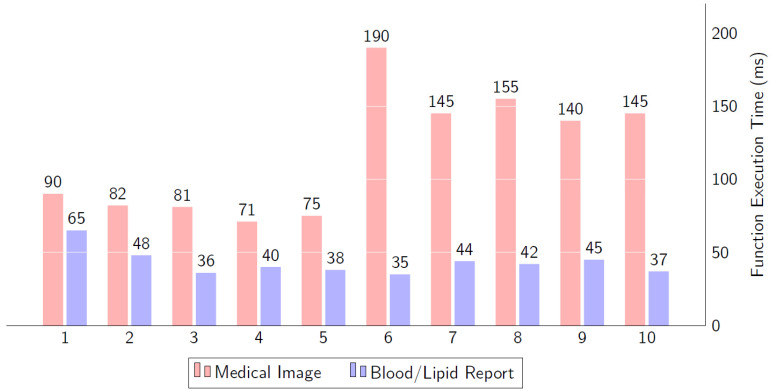
The computational time for function execution to access the data from the IPFS and blockchain.

Figure 9 presents the execution time for data accessing. The data arrive as two different types of data. The first type of data is medical images, which come from the IPFS through the file hash. However, for Number 6, the execution time is high because, here, the file size has increased; it is a big size medical image that needs more time to show or download from the IPFS. The second part is to get the textual reports of blood or lipid from the blockchain ledger.

### 5.4. Threats to Validity

A few potential research validity issues have been clearly discussed. Validity risks are limitations or restrictions that affect the design, implementation, and validation of a solution. Validity vulnerabilities must be eliminated as part of future attempts to enhance the solution and its repercussions.

Threats to Internal Validity: These refer to the restrictions that impact the proposed system’s design and implementation. For example, if medical data are utilized to execute trials in order to obtain the output, the outcome may change in terms of performance.

External Validity: External validity connects to the solution’s validation via a variety of relevant case studies and systems. We employed a case study strategy, as detailed in the research method and assessment section, to demonstrate and evaluate the answer. In the future, more case studies will be required to lessen the impacts of external validity.

## 6. Conclusions

Medical images of patients are an invaluable resource in any healthcare system’s intelligence. Ordinarily, medical images are dispersed across several systems, and sharing them is critical to developing integrated and effective healthcare. Furthermore, a centralized picture data hosting site (e.g., a cloud-assisted solution) might have the potential risk of a security breach. With the rising understanding of the dispersed nature of healthcare, decentralized designs and system interoperability have received a lot of attention. In this study, we have presented a proof-of-concept framework for a proposed PCRIM system, which is a decentralized framework based on the Ethereum blockchain and an IPFS to store and distribute medical images. The PCRIM technology enables a one-of-a-kind solution to increase patients’ rights by providing them with complete control over their medical images and medical test reports (e.g., blood reports, lipid reports) via the DApp web application portal. Patients have total control over their medical images and can issue or cancel consent to use them in academic research or clinical trials. We used an experimental demonstration to study and assess the suggested scheme’s efficiency, rationality, and practicality. While sharing access to medical pictures, the suggested system provides patients access to an immutable medical database, resulting in data provenance, increased efficiency, and effective audit. Because the exchange mechanism and data storage are decentralized, third-party middlemen or administrative organizations are not needed.

Limitations: Each transaction or transmission of medical data in the proposed system, which is based on the Ethereum platform, is subject to an Ether cost.

Future direction: In the future, our system will be further improved to the pinnacle of contemporary research with the use of a mobile-enabled smart app. For patients and clinicians to check medical reports, a smart app will be a feasible option to use. With future case studies, our prime focus will be the diversity of data evaluation, which can further increase the evaluation’s rigor.

## Figures and Tables

**Table 1:** Comparison of the proposed and existing PCRIM systems.

Scheme	MedBlock [35]	MeDShare [33]	MedChain [34]	PCIM [38]	PCRIM System
Source Data Storage	Dedicated	Cloud Server	Mutable P2P	Immutable IPFS	Immutable IPFS Storage
Server Attack Resistance	No	No	Yes	Yes	Yes
Database Management	Centralized	Centralized	Semi-Centralized	Decentralized	Decentralized
Data Access	No	No	No	Yes	Yes
Smart Contract	No	Yes	No	Yes	Yes
Patient-Centric Test Report	No	No	No	No	Yes

**Table 2:** Cost analysis of smart contract execution (gas cost = 2 Gwei).

Execution Type	Gas Used	Cost in Ether
Contract Creation	2,869,227	0.05738454
Initial Contract	225,237	0.0450474
Initial Migration Call	42,363	0.0084726
Contract Migration Call	27,363	0.0054726
Final cost		0.06188928

## Data Availability

Not applicable.

## References

[1] Casalino L., Gillies R.R., Shortell S.M., Schmittdiel J.A., Bodenheimer T., Robinson J.C., Rundall T., Oswald N., Schauffler H., Wang M.C. (2003). External incentives, information technology, and organized processes to improve health care quality for patients with chronic diseases. J. Am. Med. Assoc..

[2] DOC (U.S. Department of Commerce) (1999). The Emerging Digital Economy II: Appendices.

[3] Heart T., Ben-Assuli O., Shabtai I. (2017). A Review of PHR, EMR and EHR Integration: A More Personalized Healthcare and Public Health Policy. Health Policy Technol..

[4] Zhu L., Wu Y., Gai K., Choo K.K.R. (2019). Controllable and trustworthy blockchain-based cloud data management. Future Gener. Comput. Syst..

[5] Siyal A.A., Aisha Z.J., Muhammad Z., Kainat A., Aiman K., Georgia S. (2019). Applications of blockchain technology in medicine and healthcare: Challenges and future perspectives. Cryptography.

[6] Dai M., Zhang S., Wang H., Jin S. (2018). A low storage room requirement framework for distributed ledger in blockchain. IEEE Access.

[7] Razzaq A., Mohsan S.A.H., Ghayyur S.A.K., Alsharif M.H., Alkahtani H.K., Karim F.K., Mostafa S.M. (2022). Blockchain-Enabled Decentralized Secure Big Data of Remote Sensing. Electronics.

[8] Liu Y., Tian J., Hu R., Yang B., Liu S., Yin L., Zheng W. (2022). Improved Feature Point Pair Purification Algorithm Based on SIFT During Endoscope Image Stitching. Front. Neurorobotics.

[9] Cao Z., Wang Y., Zheng W., Yin L., Tang Y., Miao W., Liu S., Yang B. (2022). The algorithm of stereo vision and shape from shading based on endoscope imaging. Biomed. Signal Process. Control.

[10] Seh A.H., Zarour M., Alenezi M., Sarkar A.K., Agrawal A., Khan A.R. (2020). Healthcare Data Breaches: Insights and Implications. Healthcare.

[11] Mettler M. Blockchain Technology in Healthcare: The Revolution Starts Here. Proceedings of the IEEE 18th International Conference on e-Health Networking, Applications and Services (Healthcom).

[12] Halamka J.D., Lippman A., Ekblaw A. (2017). The Potential for Blockchain to Transform Electronic Health Records. Harvard Bus. Rev..

[13] Benet J. (2014). IPFS—Content Addressed, Versioned, P2P File System(DRAFT 3). https://arxiv.org/abs/1407.3561.

[14] Shini S.G., Thomas T., Chithraranjan K. (2012). Cloud Based Medical Image Exchange-Security Challenges. Procedia Eng..

[15] Perera G., Holbrook A., Thabane L., Foster G., Willison D.J. (2011). Views on health information sharing using electronic medical records. Int. J. Med. Inf..

[16] Zhang M., Chen Y., Lin J. (2021). A Privacy-Preserving Optimization of Neighborhood-Based Recommendation for Medical-Aided Diagnosis and Treatment. IEEE Internet Things J..

[17] Kish L.J., Topol E.J. (2015). Unpatients—Why patients should own their medical data. Nat. Biotechnol..

[18] Yaga D., Mell P., Roby N., Scarfone K. (2019). Blockchain technology overview. arXiv.

[19] Razzaq A. (2022). Blockchain-based secure data transmission for internet of underwater things. Clust. Comput..

[20] Makridakis S., Christodoulou K. (2019). Blockchain: Current challenges and future pro-spects/applications. Future Internet.

[21] Kakadis G., Langer S.G. (2011). Informatics in Medical Imaging: Chapter 3.

[22] Wang Y., Li P.F., Tian Y., Ren J.J., Li J.S. (2017). A shared decision-making system for diabetes medication choice. IEEE J. Biomed. Health Inform..

[23] Li C., Dong M., Li J., Xu G., Chen X.-B., Liu W., Ota K. (2022). Efficient Medical Big Data Management with Keyword-Searchable Encryption in Healthchain. IEEE Syst. J..

[24] Lee S.J., Larson E.B., Dublin S., Walker R.L., Marcum Z., Barnes D.E. (2017). Electronic medical record (EMR) predictors of undiagnosed dementia. Alzheimers Dement..

[25] Khan A.N., Kiah M.L.M., Ali M., Madani S.A., Khan A.U.R., Shamshirband S. (2014). BSS: Block-based sharing scheme for secure data storage services in mobile cloud environment. J. Super Comput..

[26] Jena D., Mishra B. Securing Files in the Cloud. Proceedings of the 2016 IEEE International Conference on Cloud Computing in Emerging Markets (CCEM).

[27] Li H., Zhao X., Wang Y., Lou X., Chen S., Deng H., Shi L., Xie J., Tang D., Zhao J. (2021). Damaged lung gas exchange function of discharged COVID-19 patients detected by hyperpolarized 129Xe MRI. Sci. Adv..

[28] O’Driscoll A., Daugelaite J., Sleator R.D. (2013). ‘Big data’, Hadoop and cloud computing in genomics. J. Biomed. Inform..

[29] The Economist Intelligence Unit of IBM Institute for Business Value (2017). Healthcare Rallies for Blockchains: Keeping Patients at the Center.

[30] Erickson B. (2011). Experience with Importation of Electronic Images into the Medical Record from Physical Media. J. Digit. Imaging.

[31] Eichelberg M., Kleber K., Kämmerer M. (2020). Cybersecurity Challenges for PACS and Medical Imaging. J. Digit. Imaging.

[32] Vest J.R., Gamn L.D. (2010). Health Information Exchange: Persistent Challenges and New Strategies. J. Am. Med. Inform. Assoc..

[33] Xia Q., Sifah E.B., Asamoah K.O., Gao J., Du X., Guizani M. (2017). MeDShare: Trust-Less Medical Data Sharing among Cloud Service Providers via Blockchain. IEEE Access.

[34] Shen B., Guo J., Yang Y. (2019). MedChain: Efficient Healthcare Data Sharing via Blockchain. Appl. Sci..

[35] Fan K., Wang S., Ren Y., Li H., Yang Y. (2018). System-level Quality Imporvement MedBlock: Efficient and Secure Medical Data Sharing Via Blockchain. J. Med. Syst..

[36] Zyskind G., Nathan O., Pentland A. Decentralizing privacy: Using blockchain to protect personal data. Proceedings of the IEEE Security and Privacy Workshops.

[37] Zhang M., Chen Y., Susilo W. (2020). PPO-CPQ: A Privacy-Preserving Optimization of Clinical Pathway Query for E-Healthcare Systems. IEEE Internet Things J..

[38] Duan C., Deng H., Xiao S., Xie J., Li H., Zhao X., Han D., Sun X., Lou X., Ye C. (2022). Accelerate gas diffusion-weighted MRI for lung morphometry with deep learning. Eur. Radiol..

[39] Jabarulla M.Y., Lee H.-N. (2020). Blockchain-based distributed patient-centric image management system. Appl. Sci..

[40] Ahmed E., Yaqoob I., Gani A., Imran M., Guizani M. (2016). Internet-of-things-based smart environments: State of the art, taxonomy, and open research challenges. IEEE Wirel. Commun..

[41] Larrucea X., Combelles A., Favaro J., Taneja K. (2017). Software engineering for the internet of things. IEEE Softw..

[42] Kitchenham B., Brereton O.P., Budgen D., Turner M., Bailey J., Linkman S. (2009). Systematic literature reviews in software engineering a systematic literature review. Inf. Softw. Technol..

[43] Manville C., Cochrane G., Jonathan C.A.V.E., Millard J., Pederson J.K., Thaarup R.K., Wik A.L., Wik M.W. (2014). Mapping Smart Cities in the EU.

[44] Estdale J., Georgiadou E. (2018). Applying the iso/iec 25010 quality models to software product. European Conference on Software Process Improvement.

[45] Truffle Suite. https://www.trufflesuite.com/guides/configuring-visual-studio-code.html.

[46] Truffle Suite. Https://truffleframework.com/docs/ganache/overview.

[47] MetaMask. https://metamask.io/.

[48] Wood G. Ethereum: A Secure Decentralised Generalised Transaction Ledger. https://gavwood.com/paper.pdf.

